# Deceptive but Not Honest Manipulative Actions Are Associated with Increased Interaction between Middle and Inferior Frontal gyri

**DOI:** 10.3389/fnins.2017.00482

**Published:** 2017-08-31

**Authors:** Maxim Kireev, Alexander Korotkov, Natalia Medvedeva, Ruslan Masharipov, Svyatoslav Medvedev

**Affiliations:** ^1^N.P. Bechtereva Institute of the Human Brain, Russian Academy of Sciences St. Petersburg, Russia; ^2^Faculty of Liberal Arts and Sciences, St. Petersburg State University St. Petersburg, Russia

**Keywords:** deception, prefrontal cortex, psychophysiological interactions, middle frontal gyrus, temporo-parietal junction, inferior frontal gyrus, social decision

## Abstract

The prefrontal cortex is believed to be responsible for execution of deceptive behavior and its involvement is associated with greater cognitive efforts. It is also generally assumed that deception is associated with the inhibition of default honest actions. However, the precise neurophysiological mechanisms underlying this process remain largely unknown. The present study was aimed to use functional magnetic resonance imaging to reveal the underlying functional integration within the prefrontal cortex during the task which requires that subjects to deliberately mislead an opponent through the sequential execution of deceptive and honest claims. To address this issue, we performed psychophysiological interaction (PPI) analysis, which allows for statistical assessment of changes in functional relationships between active brain areas in changing psychological contexts. As a result the whole brain PPI-analysis established that both manipulative honest and deceptive claiming were associated with an increase in connectivity between the left middle frontal gyrus and right temporo-parietal junction (rTPJ). Taking into account the role played by rTPJ in processes associated with the theory of mind the revealed data can reflect possible influence of socio-cognitive context on the process of selecting manipulative claiming regardless their honest or deceptive nature. Direct comparison between deceptive and honest claims revealed pattern enhancement of coupling between the left middle frontal gyrus and the left inferior frontal gyrus. This finding provided evidence that the execution of deception relies to a greater extent on higher-order hierarchically-organized brain mechanisms of executive control required to select between two competing deceptive or honest task sets.

## Introduction

Deception is an important component of human social interaction that is based on attempts to manipulate the opinions or beliefs of others. Because of its social relevance, lying is a constant focus of scientific research, with research interest recently reinforced by the rapid development of modern neuroimaging methods. A growing body of evidence from neuroimaging studies has shed light on how the brain processes deception (reviewed in Abe, 2009; Christ et al., 2009; Lisofsky et al., 2014). A majority of studies have suggested that there is a specific link between prefrontal cortex (PFC) activity and the execution of deception. It is usually noted that this assumption is in agreement with the hypothesis that deception is a process that requires greater cognitive effort and the inhibition of default honest actions. Among neuroimaging studies there is a great deal of evidence to support this view: a number of reports has demonstrated relative increases of functional activity levels in the PFC (Langleben et al., 2002; Ganis et al., 2003; Spence et al., 2004; Phan et al., 2005; Abe et al., 2006; Abe, 2009; Christ et al., 2009; Ganis and Keenan, 2009; Lee et al., 2009; Ito et al., 2011; Marchewka et al., 2012; Ding et al., 2013; Kireev et al., 2013). Areas of the middle frontal gyrus (MFG), inferior frontal gyrus (IFG), and anterior cingulate cortex (ACC) are usually associated with decision making, action inhibition and conflict monitoring are systematically revealed in experimental settings which assume execution of deception. The majority of these findings suggest that a relatively greater degree of cognitive control is applied to deceptive behavior. However, based on the experimental data it is difficult to conclude which aspects of functional activity in the PFC can be unequivocally attributed to deception. Since instructed deception is frequently used in those studies observed findings can be rather associated with an unspecific executive control process. Accordingly, in the current study deception is considered to be “a successful or unsuccessful deliberate attempt, without forewarning, to create in another a belief that the communicator considers to be untrue” (Vrij, 2008). This definition emphasizes that intentionality and manipulativeness are the key aspects of deceptive behavior.

Taking this into account, there has been a recent methodological shift toward ecologically-valid experimental designs that rely to a greater extent on free-choice conditions rather than instructed behavior (Kireev et al., 2007, 2013; Baumgartner et al., 2009; Greene and Paxton, 2009; Sip et al., 2010, 2012; Ding et al., 2013; Abe and Greene, 2014; Abe et al., 2014; Volz et al., 2015). Neuroimaging data obtained in such studies have consistently demonstrated that the PFC contributes to the execution of both deceptive and honest actions (Sip et al., 2010; Kireev et al., 2013), as well as decision making (Ito et al., 2012; Abe et al., 2014). In some cases, the execution of honest actions is also associated with a greater level of activity in the PFC, which corresponds to “difficult” decisions to refrain from lying in subjects who were prone to be deceptive to earn a higher monetary reward (Greene and Paxton, 2009). Similar increase in activity of anterior frontal gyrus, temporal gyrus and right temporo-parietal junction (rTPJ) was observed in the interactive game settings for formally honest, but behaviorally manipulative intentional actions (Volz et al., 2015). The authors called such truthful actions “sophisticated deception” (Sutter, 2009) since such honest information is sent to a receiver with an intention to mislead. Nevertheless in our previous PET-fMRI and ERP studies (Kireev et al., 2007, 2013), which utilized the same principle of interactional and manipulative game as the one used by Sutter (2009) and Volz et al. (2015), we did not reveal such differences between plain deception and manipulative honest actions. Considering the contradictory in the findings observed in the studies mentioned above the substantial diversity of experimental paradigms, statistical methods (Bayesian Volz et al., 2015 or frequentist statistics, Greene and Paxton, 2009; Sip et al., 2010, 2012; Kireev et al., 2013, etc.) and applied thresholds can be noticed. However, we believe that the methods aimed at revealing not only functional specialization but also functional integration between involved brain areas (Friston, 2011) would enable us to acquire new valuable and complimentary information regarding the reorganization of the functional interactions underlying deceptive behavior in ecologically valid settings assuming free decision making.

As a few such studies have been conducted, it is difficult to draw consistent conclusions from the reported findings. For instance, in the study utilizing functional near-infrared spectroscopy (Ding et al., 2013) a greater functional connectivity was demonstrated between the left middle frontal gyrus (lMFG) and left superior frontal gyrus during non-instructed, deceptive trials than during truthful tasks. A relatively broad degree of deceptive-task-induced functional connectivity was demonstrated using functional magnetic resonance imaging (fMRI) in areas in the frontal lobe, parietal lobe, anterior cingulate gyrus, and cerebellum (Jiang et al., 2015). To reveal the presence of a functionally connected network among these brain areas the abovementioned studies utilized correlation methods (also see Wang et al., 2015) or graph theory methods for identifying network topology (Zhang et al., 2016), therefore, these findings do not provide information how these connections vary as a function of executive actions.

The use of transcranial stimulation is another promising approach to study causal relationship between changes in brain activity related to deception. A large body of data suggests that transcranial magnetic stimulation (TMS) or transcranial direct current stimulation (tDCS) when applied to the middle, superior or inferior frontal gyri can selectively affect the speed of deceptive actions (Priori et al., 2008), improve the deceptive behavior in terms of reaction time (Karim et al., 2010; Mameli et al., 2010) or even modulate the rate of deception (Karton and Bachmann, 2011; Karton et al., 2014). However, in some instances, TMS and tDCS stimulation of the aPFC can have much less significant effects on deceptive behavior (Mameli et al., 2010; Verschuere et al., 2012).

In the majority of those studies, different parts of dorsolateral prefrontal cortex were stimulated via the electrodes placed on F3/F4 or Fp2 locations corresponded to the international EEG 10/20 system (Priori et al., 2008; Karim et al., 2010; Mameli et al., 2010). Taking into consideration that the superior and medial frontal gyri, i.e., the rostrolateral part of PFC (BA 9/10), are the main brain areas located in vicinity of the stimulated electrodes (Okamoto et al., 2004), it can be proposed that the observed behavioral effects were associated with the modulation of a number of higher-order cognitive processes associated with retaining in working memory the representations of current goals and the ways of their achievements (Nee and D'Esposito, 2016), maintenance of abstract rules of percepts-actions associations and their implementation in accordance with current context (Badre, 2008; Badre et al., 2010) or even analogical reasoning while integrating and inferring semantic relations (Bunge et al., 2009; Westphal et al., 2016), of selected manipulative actions for deceptive purposes. All these process are likely to contribute to decision making and execution of actions aimed at manipulation by an opponent which is the key feature of deception. The settings of deception are additionally complicated by possible social context as socially relevant information regarding the protagonist reputation and trustworthiness is taken into account for the purposes of effective manipulations.

Although it is generally assumed that transcranial brain stimulation influences deception-related behavior by modulating the function of these aPFC structures (Ganis, 2014; Mameli et al., 2017), the inconsistencies in behavioral effects, location of stimulated areas and used experimental tasks precludes clear understanding of exact neurophysiological mechanism underling the execution of deceptive behavior. Considering the ambiguity regarding functional role of the aPFC in the deception execution, the present study is designed to reveal changes in the functional coupling between structures of the aPFC and other brain areas involved in the execution of freely chosen deceptive and honest actions, which can shed lights on brain mechanisms and psychological operations involved in the execution of deception. According to one of the popular notions, deception is specifically associated with an action inhibition and withholding the truth is a key aspect of deceptive behavior (Verschuere et al., 2012; Debey et al., 2015). Specifically lying is considered as a two-step process including the activation of honest representation and its subsequent inhibition (Debey et al., 2014). Based on the notion that operation of response inhibition is tightly associated with the activity of the right inferior frontal gyrus (Aron et al., 2014) it can be predicted that aPFC will greater interact with the right IFG for deceptive actions then for honest ones. The impact of the right IFG to truth suppression was demonstrated in a number of previous studies (Abe et al., 2006; Bhatt et al., 2008). The direct link between elevated local activity within the right IFG and lying was demonstrated by Vartanian et al. (2012) who argued that activity in this area predicts successful lying and is associated with effective inhibitory control.

On the other hand, it is hard to differentiate an action inhibition from an action selection (Mostofsky and Simmonds, 2008), since these operations act synergistically for selecting goal-relevant actions rather than represent separate and independent operations. To deceive one has to retain relevant information about current goal and context in working memory: task instructions, stimulus-response mapping and recent history of feedback from a partner, i.e., an abstract rules that guide the selection of representation of particular action. It is largely accepted that the more abstract rule is applied the more anterior part of the PFC is involved (Koechlin et al., 2003; Badre, 2008). Accordingly deception execution can be relied on a general ability of response selection needed for selecting a representation of a goal-appropriate response within the same task set. Taking into account the experimental data evidencing that the left IFG can play a key role in operations of response selection (Moss et al., 2005; Goghari and MacDonald, 2009) and, it can be expected that deceptive actions will lead to enhancement of interaction between the rostrolateral aPFC and the lIFG. Additionally we checked another possibility derived from the studies of Sutter (2009) and Volz et al. (2015) who suggested that honest acting for misleading purposes can be considered as sophisticated deception, which in comparison with plain deceptive claims can at a greater extend involve the processes of mentalizing about protagonist intentions to accept or refuse a claim.

In order to test these assumptions we utilized psychophysiological interaction analysis (PPI; Gitelman et al., 2003) methods on fMRI data obtained in our previous study, which modeled an interactive sender-receiver game with a computer opponent (Kireev et al., 2013). In comparison with conventional analyses of changes in the levels of functional activity, the advantage of PPI analysis lies in the ability to reveal functional integration in a manner that is independent of variability in local activity levels. In the present study, PPI analysis is expected to provide insight into how differences between freely-chosen honest and deceptive claims modulate the functional coupling between two analyzed brain regions. Based on the known role of the PFC in executive and cognitive control, as well as experimental data demonstrating how TMS and tDCS applied to the aPFC affect the execution of deception, the current PPI-data analysis will be focused on the identifying changes of functional interactions between the aPFC and all the other voxels of the brain.

## Materials and methods

### Participants

Twenty-four healthy volunteers (14 women and 10 men) participated in the study. All participants were native Russian speakers, 19–44 years of age, with no history of neurological or psychological disorders. All subjects were also right-handed, as assessed by the Edinburgh Handedness Inventory (Oldfield, 1971). The participants were given no information about the specific purpose of the study. All subjects provided written informed consent prior to the study, and were paid for their participation. All procedures were conducted in accordance with the Declaration of Helsinki and were approved by the Ethics Committee of the N.P. Bechtereva Institute of the Human Brain, Russian Academy of Sciences.

### Study design

**Figure 1:**
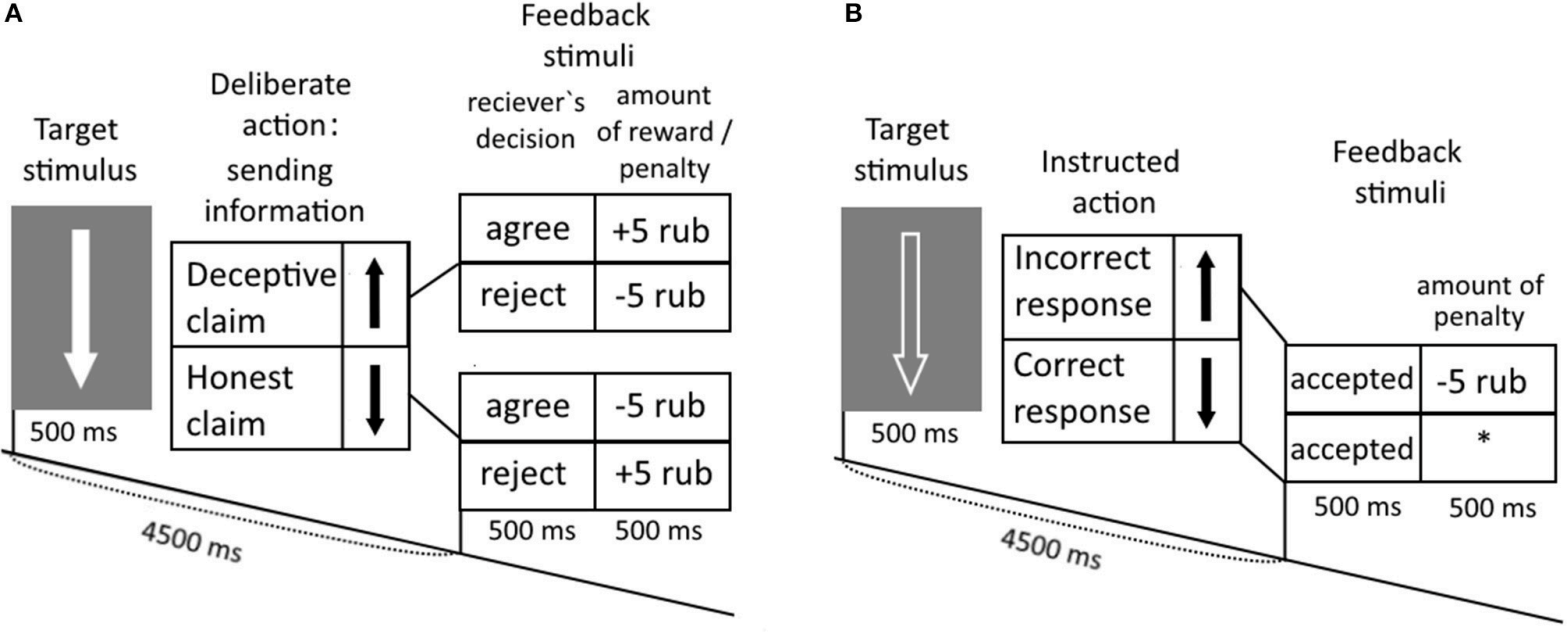
Experimental task. **(A)** Subjects were instructed to interact with the opponent by falsely of honestly claiming about an arrow direction of the solid arrows presented on the monitor. **(B)** In response to the edged arrows, subjects were instructed to press buttons in accordance with the direction of the arrows.

Subjects played an interactive game with the computer opponent, which utilized the principle of “I doubt it” card game. They were instructed to send to the computer opponent false or truthful information regarding the direction of presented up- and down-ward arrows (Kireev et al., 2007, 2013). The primary goal of the experimental task was to mislead the computer opponent in trial by trial manner. Before the study each participant was instructed that: (1) she plays with a special computer program, which tries to predict the trustworthy of claims received from her; (2) after the “decision” to accept or reject a claim computer acquires the information about the real arrow direction and correspondingly adopts its tactics in future trials. Participants were able to freely choose to lie or be honest. Claims were provided by pressing corresponding buttons with fixed relation between particular button and sent direction. In each trial, the computer decided to accept or refuse received claims entered by subjects via presenting corresponding feedback stimuli on the screen. There were three types of experimental trials: honest claims (HC), deceptive claims (DC), and control catch trials. Catch trials served to control the subject's awareness about association between button and arrow direction and consisting of button-pressing behavior in strict accordance with the presented stimuli). In each trial “win,” “defeat,” and control feedback stimuli were presented to provide subjects with information regarding the “decision” of the computer opponent to accept or refuse their claims (See Figure 1). Subjects were monetarily rewarded for accepted-deceptive or challenged-truthful claims, and penalized in accepted-honest and refused-deceptive claims. At the end of the trial a corresponding monetary reward was presented, just after the feedback stimuli was presented from the computer informing subjects of the “receiver's decision.” Incorrect button pressing in Catch trials was also penalized.

Indeed subjects were misled regarding gaming since feedback stimuli were randomly presented. However, based upon participant's queries, they tried to manipulate the computer's “decisions” about their use of honest and deceptive claims. To induce deceptive claiming the word “agree” was presented in 60% of trials. Such percentage was empirically defined in behavioral study before our first ERP study utilizing this paradigm (Kireev et al., 2007, 2008) and allowed to reach balanced ratio between honest and deceptive claims (see also Kireev et al., 2013).

The study consisted of two experimental runs, 15 min in duration each. Sixty gaming trials with deceptive or truth claims were intermixed with Catch trials (Figure 1B). The outlook of the presented arrow was used as a cue for gaming and control trials. The use of a white, solid arrow, or black edged arrow with a white outline, respectively. In a gaming trials a subject sent truthful or false information regarding an arrow orientation to an opponent. To control correct stimulus-response mappings in the catch trials the subjects pressed buttons in accordance with the direction of the presented arrows.

### Data acquisition and preprocessing

Magnetic resonance imaging was performed using a 3 Tesla Philips Achieva (Philips Medical Systems, Best, The Netherlands). Structural images were acquired using a T1-weighted pulse sequence (T1W-3D-FFE; repetition time [TR] = 2.5 ms; TE = 3.1 ms; 30° flip angle), measuring 130 axial slices (field of view [FOV] = 240 × 240 mm; 256 × 256 scan matrix) of 0.94 mm thickness. Functional images were obtained using an echo planar imaging (EPI) sequence (TE = 35 ms; 90° flip angle; FOV = 208 × 208 mm; 128 × 128 scan matrix). Thirty-two continuous 3.5-mm-thick axial slices (voxel size = 3 × 3 × 3.5 mm), covering the entire cerebrum and most of the cerebellum, were oriented with respect to the structural image. The images were acquired using a TR of 2,000 ms. Image pre-processing and statistical analyses of the fMRI data were performed using SPM12 software (Statistical parametric mapping, http://www.fil.ion.ucl.ac.uk/spm/software/spm12/). The data obtained for each subject were spatially realigned to the first functional image. To avoid effects from differences in the time of acquisition for each slice, slice-time correction was applied. The resulting functional images were spatially normalized to a standard stereotactic MNI template (Montreal Neurological Institute) and smoothed (using a Gaussian filter, 8 mm full-width at half-maximum). To prevent head motions of participants “Philadelphia” cervical MRI-compatible collar was used.

#### Analysis of psychophysiological interactions

To test hypotheses regarding action inhibition and action selection operations involved in deception, we selected region of interest (ROI) by calculating conjunction contrast for DC>Catch and HC>Catch (Kireev et al., 2013) which revealed a pattern of common brain areas involved in both deceptive and the honest manipulative claims including and frontal and parietal brain areas usually observed in fMRI/PET studies of deception. Taking into account that the paradigm of the current study assumes free decision making in the settings of uncertainty which action to select, we purposed that the key aspect of action execution in these settings is associated with application of abstract rules governing purposeful behavior which is supported by a rostrolateral part of aPFC (Badre, 2008; Badre et al., 2010). The activation within the anterior frontal cortex associated with the execution of the both deceptive and honest claims was found in the lMFG (BA10), which was used in the current PPI analysis. This area has been frequently observed in neuroimaging studies of deception and activity in this region has been attributed to processes underlying the execution of deception (Ganis et al., 2003; Nuñez et al., 2005; Abe et al., 2008), decisions to lie (Greene and Paxton, 2009; Sip et al., 2010) preparation for both deceptive and honest actions (Ito et al., 2012) as well as sophisticated deception (Volz et al., 2015).

In the present study, context-dependent whole-brain changes in functional coupling were therefore calculated for a region comprising a 4-mm-radius sphere located in the lMFG (BA10) (coordinates *x* = –39, *y* = 53, *z* = 1), located close to the ROI analyzed in Westphal et al. (2016) study where the role of this region in analogical reasoning was demonstrated. For the PPI-analysis we applied the generalized PPI toolbox (gPPI; McLaren et al., 2012; [http://www.nitrc.org/projects/gppi)]. Briefly, this approach allows for the statistical assessment of the extent of the relationship between the neuronal activities resulting from experimental manipulations in predefined ROIs, as well as in other brain regions (either voxels or other ROIs). In contrast to conventional PPI analysis (as implemented by SPM12 software), gPPI allows for the separate calculation of regression coefficients for all experimental events of interest in a general linear model (GLM). In the present study, PPI-predictors were created based on the blood oxygen level-dependent (BOLD) time series data that were extracted from selected ROI in the lMFG, in accordance with the following standardized procedure: (1) at the deconvolution step, the neuronal activity underlying the observed BOLD changes within ROI was mathematically estimated (Gitelman et al., 2003); (2) estimated parameters for neuronal activity were then multiplied by vectors describing experimental “on-times” that corresponded to events of interest with zero durations (i.e., instances of button-pressing in DC, HC and correct or incorrect responses during Catch trials, and feedback stimuli); and (3) resulting vectors were subsequently convolved with a hemodynamic response function (see McLaren et al., 2012; Cisler et al., 2013). In addition to PPI-predictors (i.e., psychophysiological interaction terms), the GLM contained the following regressors, which were used as ignored variables: (1) six regressors that modeled BOLD signal changes induced by DC, HC, Catch and subsequent feedback stimuli (such as those in conventional subtractive GLM analysis, typically described as psychological variables); (2) trials without responses and wrong button presses in Catch trials; (3) motion parameters; and (4) the time series corresponding to BOLD signal changes within the lMFG ROI to exclude context-dependent hemodynamic changes. Both PPI and BOLD regressors (used as ignored variables in the currents analysis) were modeled with zero durations.

To test our hypotheses at the group level we applied one way F-contrast of PPI parameters (Catch>baseline) < (HC>baseline) < (DC>baseline) which models a parametric linear modulation of functional interaction reflecting an increment of efforts need for action inhibition/action selection processes. The results of calculation of corresponding t-contrasts at the first level analysis (i.e., DC>baseline, HC>baseline, Catch>baseline) were used as variables for this group level analysis and the −0.5, 0, 0.5 vector was applied for the F-contrast estimation. We assumed that the functional coupling with the lMFG area would increase from Catch to HC and further to DC conditions. Alternatively, it could be expected that HC would be associated with a greater functional integration based on the previous fMRI-study by Volz et al. (2015), considering honest claiming for manipulative purposes as a sophisticated deception (Sutter, 2009). In order to check this assumption we calculated a subsidiary one way ANOVA F contrast (Catch>baseline) < (DC>baseline) < (HC < baseline).

The estimated beta coefficients of the corresponding PPI-predictors were calculated for every subject (i.e., a t-contrast between each of the analyzed experimental trial and baseline) and they were subsequently submitted to a second-level group analysis performed using one-way ANOVA (as implemented in SPM12 software http://www.fil.ion.ucl.ac.uk/spm/software/spm12/). To avoid false-positive findings, we applied the FWE (*p* < 0.025) correction for multiple comparisons at the cluster level with cluster defining threshold (CDT) *p* = 0.001. As it was shown in a recent study (Eklund et al., 2016) in which the rates of false positives revealed as a result of application of different fMRI-data analysis packages for FWE correction at the cluster level were compared, CDT *p* = 0.001 applied for the random event related design was characterized by relatively lower false positive rates when the SPM was used for the data analysis.

Additionally in order to directly assess the difference in the functional coupling between deceptive and honest claims, the t-contrast of DC > HC comparison was calculated in the same way. As far as pair-wise comparisons were made (i.e., DC > HC, HC > DC) to avoid false positive findings, since the same analysis was performed twice for the same data, we used the Bonferroni correction, which resulted in a cluster threshold of FWE *p* < 0.025.

The anatomical locations of the identified changes in functional integration were identified using the xjView toolbox (http://www.alivelearn.net/xjview). The REX toolbox (http://www.nitrc.org/projects/rex/) was used to demonstrate the difference between beta values in identified clusters of functional interactions changes. MRIcroN was applied for illustration of revealed clusters over standardized brain template ([http://www.mccauslandcenter.sc.edu/mricro/mricron/]).

## Results

### Behavioral results

**Figure 2:**
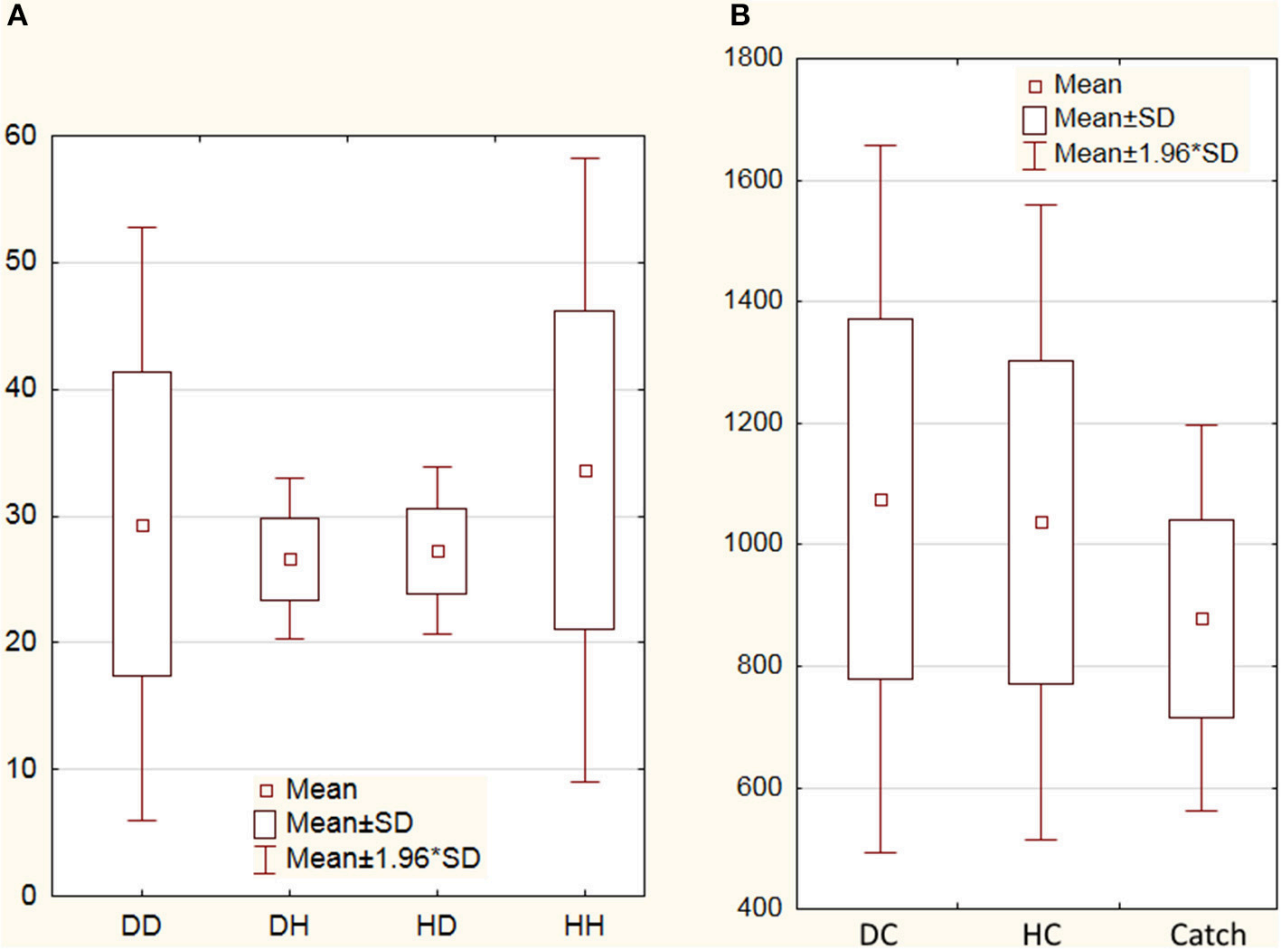
Number of deceptive and honest claims and reaction times. **(A)** Averaged numbers of deceptive and honest claims performed by subjects: DD, deceptive claims after preceding deceptive trial; DH, honest claims after deceptive trial; HD, deceptive claims after preceding honest trial; HH, honest claims after honest trial; **(B)** Averaged reaction times (in ms) for deceptive claims (DC), honest claims (HC), and Catch trials. SD denotes standard deviation.

Nonparametric Wilcoxon match pairs test did not reveal significant difference in overall group rates of deceptive [56 ± 12(SD)] and honest [61 ± 11.5(SD)] claims (*Z* = 0.8, *p* = 0.42). Nonparamentric statistical assessment of possible strategic pattern of claiming elicited significant effect of sequence of participant claims (Friedman ANOVA Chi Square test *p* = 0.04). The ratio between number of honest and deceptive claims after honest trials was greater than the ratio between deceptive and honest claims after deceptive trials (see the Figure 2A). Friedman ANOVA Chi Square test of reaction times (RTs) associated with deceptive and honest claims revealed statistically significant (*p* < 0.001) increase of RT for DC [1,067 ms ± 297(SD)] and HC [1,037 ms ± 267(SD)] trials in comparison with Catch trials (878 ms ± 162, see Figure 2B). Direct comparison between DC and HC RTs also revealed significant difference (Wilcoxon Matched Pairs Test *Z* = 2.51, *p* = 0.012).

### PPI results

**Figure 3:**
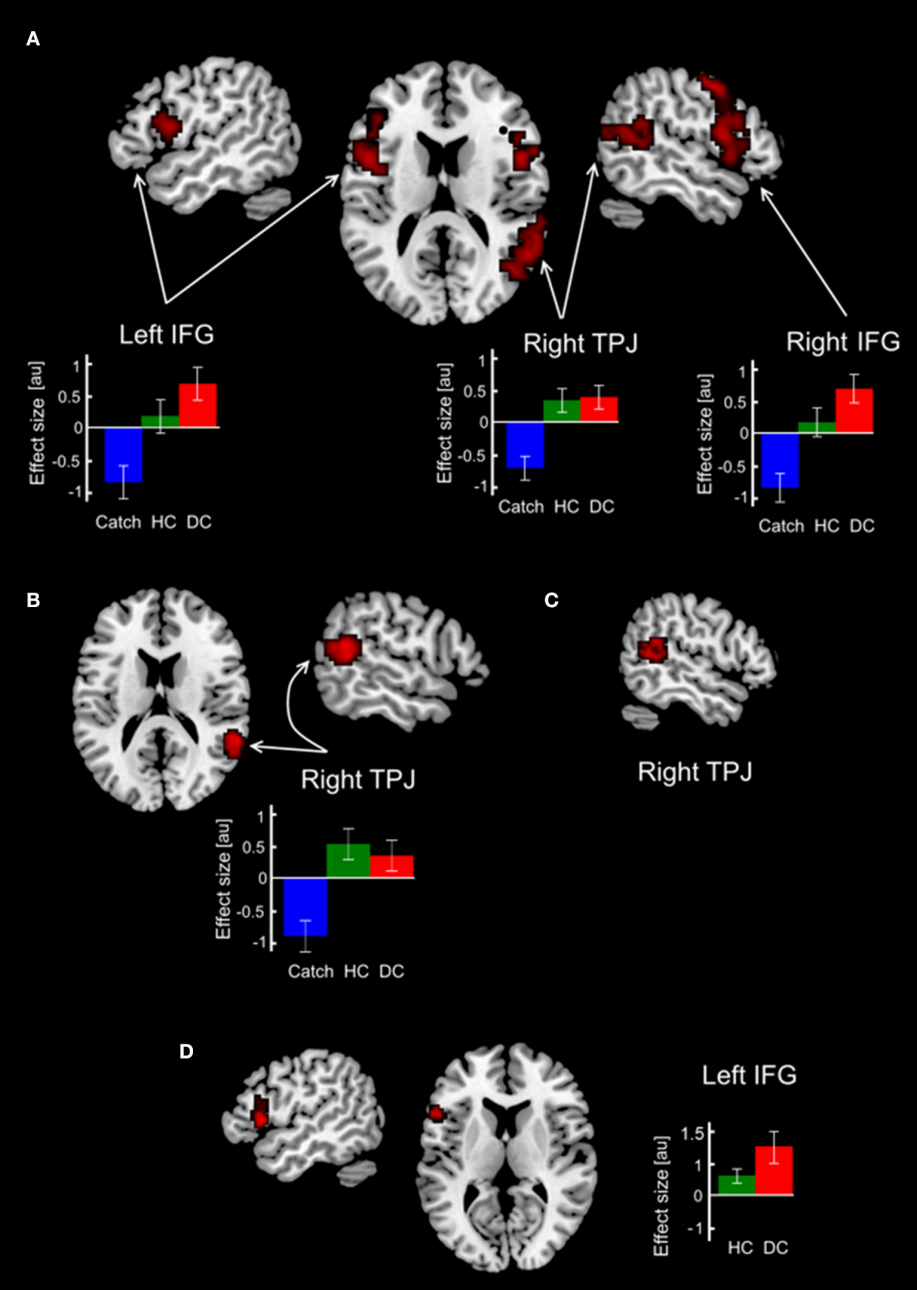
Changes in functional coupling with ROI in the lMFG. Clusters with increased functional connectivity with the lMFG overlaid on the standard 3-D brain template. **(A)** Clusters revealed in the parametric F-contrast Catch < HC < DC. **(B)** Clusters revealed in the parametric F-contrast Catch < DC < HC. **(C)** Conjunction between F contrasts in **(A,B)**. **(D)** Results of direct comparison between deceptive and honest claims. Colored bars depict PPI-parameters associated with DC, HC and Catch trials. Abbreviations: DC, HC, Catch denotes trials with deceptive claims, honest claims and Catch trials, respectively; IFG, inferior frontal gyrus; MFG, middle frontal gyrus; au, arbitrary units.

In order to test our predictions regarding involvement of brain mechanisms in the deception execution we calculated parametric F-contrast which modeled parametric modulation of functional interactions with lMFG associated with action selection/action inhibition processes which were putatively involved in HC and DC trials. Our first expectation that these processes are greater involved in deceptive claiming were supported by the obtained results (Table 1, Figure 3A). Three clusters of increased psychophysiological interactions were revealed in the lIFG, rIFG, and the rTPJ. Corresponding F-contrast modeling parametric linear modulation of functional coupling revealed an increase of interaction between lMFG and rTPJ for both HC and DC trials (Table 1, Figure 3B). Taking into account that both revealed rTPJ clusters were substantially overlapped we performed a conjunction analysis, which identified cluster of increased PPI parameters in the rTPJ (Table 1, Figure 3C) shared by both parametric F-contrasts (HC and DC trials).

**Table 1:** Analysis of functional coupling changes revealed for ROI seed in the left middle frontal gyrus (lMFG).

**Brain region**	**Cluster level pFWE**	***k***	**Peak MNI coordinates**		
			***x***	***y***	***z***
**1. F-CONTRAST CATCH < HC < DC**					
Left IFG/ Rolandic operculum/ Precentral gyrus (BA 44/45)	< 0.001	146	−48	8	16
Right IFG /Precentral gyrus (BA 44/45)	< 0.001	378	60	14	28
Right MTG/STG/IPL (BA 22/39/40)	< 0.001	222	57	−55	16
**2. F-CONTRAST CATCH < DC < HC**					
Right MTG/STG/IPL (BA 22/39/40)	< 0.001	129	57	−46	10
**3. CONJUNCTION BETWEEN CATCH < HC < DC AND CATCH < DC < HC CONTRASTS**					
Right MTG/STG/IPL (BA 22/39/40)	0.002	103	60	−49	19
**4. T-CONTRAST DC>HC**					
Left IFG (BA 44/45)	0.013	69	−51	17	14

*BA, approximate Brodmann‘s area; L/R, left/right hemisphere; k, cluster size in voxels; IFG, inferior frontal gyrus; MTG, middle temporal gyrus; STG, superior frontal gyrus; IPL, inferior parietal lobule*.

**Figure 4:**
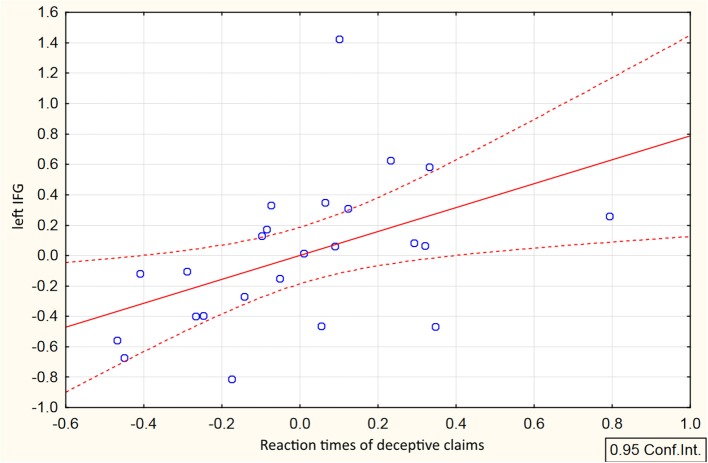
Correlation between RTs and connectivity parameters in the lIFG associated with deceptive claiming. Scatterplot represents relationship between mean-centered RTs and mean-centered first eigenvariate extracted from the cluster in the lIFG revealed in DC>HC t-contrast.

Direct comparison between HC and DC trials revealed cluster demonstrating changes in context-dependent functional coupling between the ROIs located in the left MFG located in the left inferior frontal gyrus (see Table 1, Figure 3D). No significant differences in voxels were observed for the reverse contrast (HC > DC). To illustrate differences between compared measures of psychophysiological interactions, within subject group analyses were conducted for PPI-parameters (beta values) averaged over all voxels in the identified cluster. The resulting analysis demonstrated that execution of both the deceptive and honest claims were characterized by enhanced functional integration between the lMFG and lIFG. However, greater increases in functional coupling were associated with deceptive compared with truthful trials (Figure 3D). Correlational analysis between deceptive RTs and the first eigenvariate extracted from observed cluster in the lIFG revealed positive correlation between reaction times and the PPI parameters (*r* = 0.48, *p* = 0.018, see Figure 4).

## Discussion

In the present study, subjects were instructed to make claims with an aim to mislead their computer opponent by encouraging this opponent to accept deceptive, or reject truthful claims. Both types of actions were designed to be executed with a “malicious intent” and a process of choice between alternative claims should at least partly rely on predictions of an intention of the opponent to accept or reject a proposed claim. A closer look at the averaged amounts of deceptive and honest claims indicated the lack of preference for a particular type of action. Likewise there was no particular pattern of sequence of claiming, i.e., the number of deceptive claims after honest ones did not differ significantly from the quantity of honest claim after deceptive trials. Although the difference between RT of DC and HC was not very large, revealed slowing down in RT for deceptive claims corresponded to the results of a recent meta-analysis (Suchotzki et al., 2017) demonstrating enhanced cognitive cost of deception.

Revealed PPI data showed that both honest and deceptive claiming were associated with increased functional coupling between the lMFG and the rTPJ (Figures 3A–C). Basically, the experimental settings of the current study were very similar to interactive game conditions reported in the study by Volz et al. (2015) where truthful claiming, but not deceptive, was associated with involvement of the rTPJ, one of the key brain areas responsible for inferring mind states of others in settings of social interaction (Saxe, 2009). This finding is in line with an idea that honest acting for manipulative purposes to mislead an opponent can be considered as “sophisticated deception” (Sutter, 2009). A similar situation definitely occurred in the current study, since in some trials a sender assumed that the receiver would reject his/her honest claim. But, the results of the conjunction analysis demonstrated the common cluster for the deceptive and honest claims in the rTPJ. This suggests that socio-cognitive processes associated with thinking about beliefs and expectations of others, i.e., processes related to the “Theory of Mind” (Saxe, 2009; Mar, 2011), are equally important for misleading actions irrespectively of whether deceptive or honest actions are implemented. Indeed a large body of studies showing that changes of human behavior may relate to changes of rTPJ activity in experimental settings assuming inferring intentionality of interacting agent, thinking about mental state of an opponent or acting in accordance with an opponent way of responding, i.e., social context. The link between rTPJ activity and processing of socially significant context was demonstrated in a number of meta-analyses and reviews (Carter and Huettel, 2013; Lee and Harris, 2013; Krall et al., 2015) stressing that by virtue of rTPJ activity decision making process can be modulated by the socially relevant information.

The specificity of the rTPJ activity to bluffing in the context of social interactions was demonstrated also by Carter et al. (2012). In their study, subjects played a modified poker game and authors using the combinatorial multivariate pattern analysis technique demonstrated that the activity within the rTPJ exhibit sensitivity to perceived behavioral relevance of a human opponent. Although in the present study participants interacted with non-human computer opponent, one can conclude that the rTPJ still involved in manipulating by interlocutor ability to recognize deception or misleading honest claims. This is partly supported by observed ratio between sequences of DC and HC after deceptive or honest trials (Figure 2A). Current PPI-findings also corresponded to the results of the recent study measuring BOLD signal changes associated with interactive gaming with human, human-like, non-human-like robots, and computer opponents (Suzuki et al., 2014). Authors revealed the dependency between activity of the rTPJ and impression caused by a type of an opponent. Specifically, when playing with computer in a competitive game subjects tried to cover their strategy by increasing randomness of their choices to avoid reading their responding by a computer algorithm. The same can be the true for the current research, not least of all, since behavioral analysis did not reveal any preferences either in the quantity of deceptive/honest claims or their specificity of their sequences. Likewise the revealed involvement of rTPJ can be interpreted as a reflection of possible modulation of action selection by social context raised as a result of possible humanization of computer opponent (Suzuki et al., 2014). In this respect the observed increment of functional coupling between lMFG and rTPJ for both honest and deceptive claims may demonstrate manipulative purpose of their execution and extend previous fMRI findings regarding the role of rTPJ in impact of socio-cognitive processes in deception (Lisofsky et al., 2014).

Although there is an ambiguity of PPI-analysis interpretation (Friston et al., 1997), the influence of socio-cognitive context can be manifested as modulatory influence of the rTPJ activity on the prefrontal brain regions associated with control of action execution and decision making. For example, Hare et al. (2010) showed the interaction between degree of willingness to give the amount of donated money was underlined by indirect influence of rTPJ via IFG on the activity of ventromedial prefrontal cortex considered as a part of value processing brain system.

Along with that, there were context dependent changes in functional coupling specifically related to deceptive claims that were observed between the lMFG and the left and right IFG (Figure 3A). The revealed pattern of an increased functional interaction supported our proposition regarding a greater involvement of action selection/action inhibition processes in execution of deceptive claims rather than in manipulative honest claims as it could be expected from fMRI investigation by Volz et al. (2015). In the present study based on the interactive game subject's purposeful behavior involves making a conscious effort to adhere to a main goal (to defeat an opponent), information maintaining in working memory about possible outcomes of the trials, predicting the opponent's intentions to accept a claim and choosing between two possible options: truthful or false claims. According to previous reports, activity in the lMFG resulting from deception tasks was attributed to general cognitive functions such as working memory, inhibitory control, task switching, and generation of deception (Abe et al., 2006; Christ et al., 2009; Sip et al., 2010; Ito et al., 2011; Vartanian et al., 2013). In comparison, involvement of the IFG in the execution of deception is usually associated with the cognitive functions of executive control (i.e., suppression of honest actions) (Spence et al., 2008; Lee et al., 2009), action selection (Langleben et al., 2005), and task switching (Christ et al., 2009; Fullam et al., 2009). Our data therefore suggest that the interplay occurs between the maintenance of goals in working memory and cognitive control during deceptive action execution. More specifically, the observed changes in functional coupling between the lMFG and the left and right IFG point out that both action selection and response inhibition may play an important role in deception.

Taking into account the central role played by the rIFG area in action inhibition (Aron et al., 2014) it was expected that interaction with right IFG will be observed for deceptive claiming. And although some authors claimed that action inhibition is key process involved in deception (Verschuere et al., 2012; Debey et al., 2014, 2015), revealed enhancement of functional interaction between the lIFG and the lMFG elicited by direct DC>HC comparison (Figure 3D) corroborates alternative suggestion of a greater involvement of action selection rather than action inhibition processes. This proposition was supported by both slowing of RTs of deceptive claiming, as compared to honest one, and their positive correlations with PPI parameters in the revealed lIFG cluster (Figure 4). The question as to why the execution of deception compared with that of honesty requires greater functional interaction within the PFC, even under otherwise controlled conditions, remains ambiguous. A possible explanation for the unique functional correlates of deception is that task-sets for deceptive and honest actions differ at the stimulus-response level of representation. Specifically, deceptive claims are incompatible in terms of stimulus-response mapping, which is detected by action-monitoring brain mechanisms such as the error-detection system (Bechtereva et al., 2005; Kireev et al., 2013). In contrast, honest claims were relatively easy to execute because they can be partially externally driven. For instance, to press the button in accordance with stimulus orientation can enhance the selection of an honest action. This possibly explains the observed slowing in RT and greater connectivity PPI-values associated with greater deceptive RTs. Consequently, the selection of false claims assumes the resolution of discrepancies between the target stimulus and response, resulting in greater control by the aPFC. Accordingly, there are two possibilities of underling neurophysiological mechanism providing observed lMFG-lIFG coupling: (1) to execute the deceptive claims one needs to overcome this incompatibility, which is subserved by top-down modulation of the lIFG activity involved in action selection exerted form the lMFG which is responsible for the implementation of an abstract rule; (2) deceptive claiming are underlined by stronger effective interactions between this brain structures.

The fact that deception was characterized by enhancement of the functional interaction between the lMFG and the lIFG is also in accordance with the model that the rostrocaudal functional organization of the PFC and associated response-selection processes are guided by higher order behavioral goals (Koechlin and Summerfield, 2007; Badre, 2008; Blumenfeld et al., 2013; Domenech and Koechlin, 2015). The rostral regions of the PFC are specifically associated with maintaining the representations of an overarching goal or abstract task rules, while caudal regions are responsible for the concrete representation of sub-goals and action selection. Because of the free decision making paradigm utilized in our study, there were no fixed stimulus-response-outcome associations. In each trial, the subject selected between two options: to be honest or to lie. However, both claims could potentially led to a positive or negative outcome. This process of selection can be guided by the abstract representation of goal-relevant information, which comprises constant updates of abstract rules based on the processing of outcome and overall gaming efficacy. Although PPI analysis does not allow for the inference of causality in functional connections, it can be hypothesized, but not confirmed, that the MFG is dominant to the IFG in the process of task-set selection (Koechlin et al., 2003). Thus revealed lMFG-lIFG interaction can reflect the interactive relationship between goal representation and the presence of options in a behavioral task requiring the execution of sequential honest and deceptive purposeful actions. In this respect, the obtained PPI data provide an alternative account to the widespread notion that the required inhibition of default honest actions makes deception a more cognitively demanding process, but future research is needed to clarify this issue.

## Conclusions

The present PPI study demonstrated how brain network comprising deception-related brain areas behaves depending on psychological context of freely chosen honest and deceptive actions. Generally, misleading the opponent by implementing either deceptive or honest claims, while expecting that the opponent would not believe in honest one and trust in deceptive, was associated with an increased interaction between lMFG and rTPJ area. The involvement of rTPJ demonstrated possible recruiting of ToM-related processes associated with socio-cognitive context of manipulative claiming regardless their honest or deceptive nature. When compared with honest manipulative actions deceptive claims were characterized by relatively greater increase in the functional interactions between the left MFG and left IFG. This finding supported the idea that the selection between competing honest and deceptive task-sets plays a greater role in deceptive behavior then the process of action inhibition. The observed functional interaction between the rostral and caudal parts of the PFC demonstrates the effect of application of abstract task rule, which guides the action selection process.

## Author contributions

SM, MK designed the study and formulated the concept of the project. RM, NM, AK collected the data, performed connectivity analysis (PPI) and drafted the initial version of report. All authors contributed to writing, reviewing and editing of the report.

### Conflict of interest statement

The authors declare that the research was conducted in the absence of any commercial or financial relationships that could be construed as a potential conflict of interest.
